# Cerebral venous sinus thrombosis with an acute subdural hematoma treated with endovascular intervention: A case report

**DOI:** 10.3389/fneur.2022.952187

**Published:** 2022-10-28

**Authors:** Miranda Crouch, Nathan Quig, Edward Yap, Winnie Lau

**Affiliations:** ^1^Department of Neurology, University of North Carolina Hospitals, Chapel Hill, NC, United States; ^2^Department of Neurosurgery, University of North Carolina Hospitals, Chapel Hill, NC, United States

**Keywords:** sinus thrombosis, subdural hematoma, thrombectomy, coagulopathies, COVID-19

## Abstract

We report two cases of endovascular intervention for management of cerebral venous sinus thrombosis complicated by an acute intracranial hemorrhage during treatment with therapeutic anticoagulation. The first patient developed an acute subdural hematoma with progressive enlargement and was subsequently managed with venous sinus thrombectomy. The second patient developed an intraparenchymal and subdural hematoma and was treated with middle meningeal embolization. Anticoagulation is the primary treatment for cerebral venous sinus thrombosis but also contraindicated in an acute intracranial hemorrhage. In these cases, after endovascular intervention both patients resumed therapeutic anticoagulation without further hematoma expansion or additional invasive interventions. Both patients made an excellent neurological recovery and returned to their baseline functional independent status. Given the need for anticoagulation, endovascular intervention in the form of thrombectomy or middle meningeal artery embolization may be a viable adjuvant to anticoagulation in select patients.

## Introduction

Cerebral venous sinus thrombosis (CVST) is a rare cause of stroke, which is one of the leading causes of long-term morbidity and mortality (1). CVST frequently affects young adults and pre-menopausal females three times more than males, occurs more frequently in prothrombotic states such as Factor V Leiden mutation, antithrombin III deficiency, oral contraceptive, trauma, malignancy, and manipulation in operative procedures (1, 2). The cornerstone treatment for CVST is prompt initiation of anticoagulation therapy (3, 4). CVST can propagate into large cortical veins resulting in a physiological increased venous and intracranial pressure (ICP) leading to cerebral edema, hemorrhage, or a venous infarct (1, 2).

There have been a few cases reported in which patients have developed an acute subdural hematoma (aSDH) secondary to CVST (5–7). As a result, anticoagulation is a relative contraindication in these patients leaving limited treatment options for the management of CVST complicated by aSDH. Various studies have reported middle meningeal artery (MMA) embolization as a means to prevent recurrent rupture of the neovasculature supplying the SDH (8, 9).

We present two cases where endovascular interventions, venous sinus thrombectomy and MMA embolization, were utilized to manage hemorrhagic complications in patients with CVST requiring anticoagulation.

## Case reports

### Case 1

**Figure 1:**
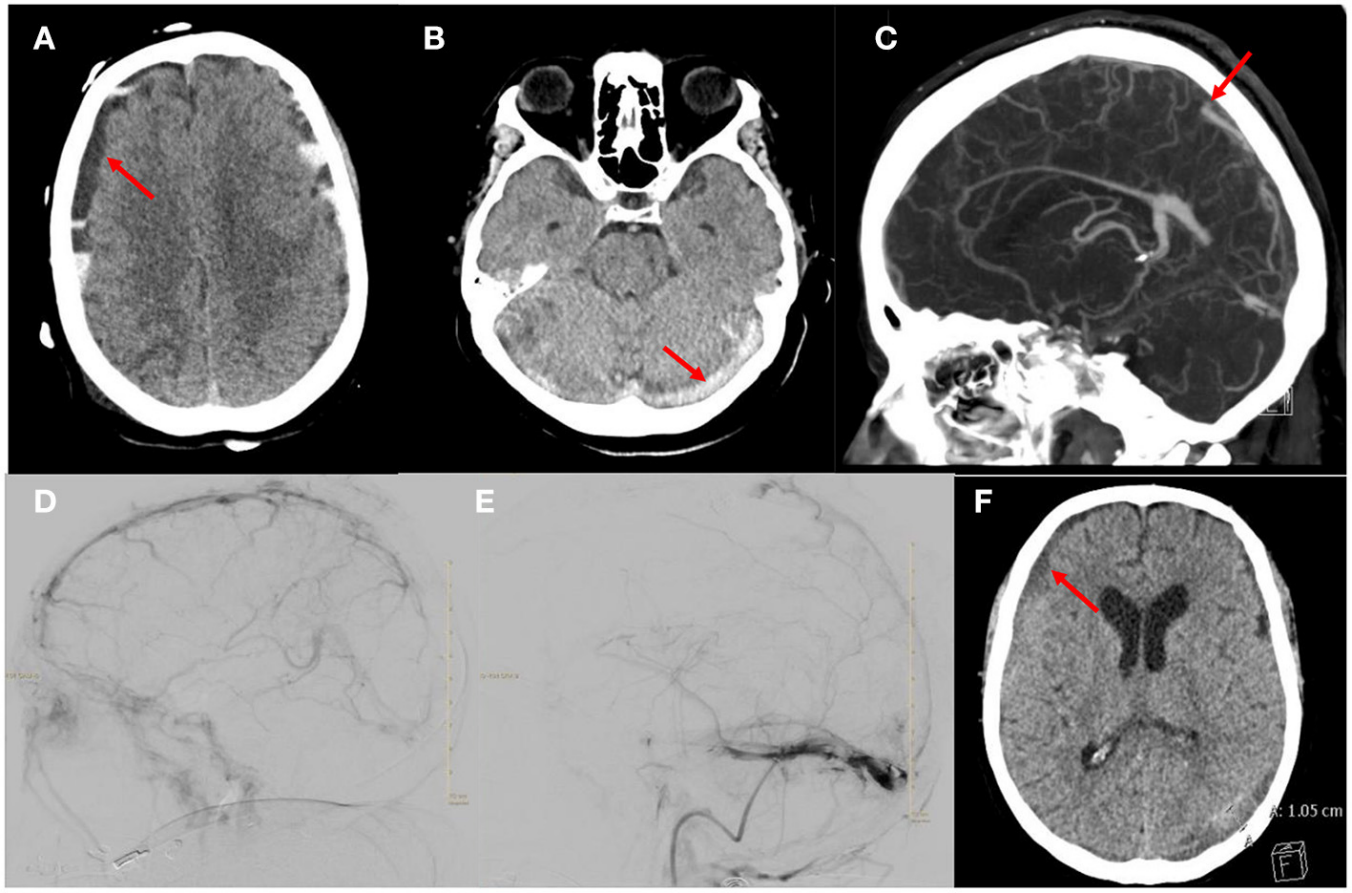
Images from the patient who received mechanical thrombectomy and anticoagulation for aSDH with CVST. **(A)** Axial head CT demonstrating the mixed density acute subdural hematoma. **(B)** Axial head CT demonstrating hyperdensity in the torcula and transverse sinuses suggestive of acute thrombosis. **(C)** Sagittal CTV demonstrating occlusion of the SSS, straight sinus and torcula along with generalized venous congestion. **(D)** Right ICA injection, lateral projection of late venous phase demonstrating thrombus in the SSS and partial occlusion of the straight sinus and occlusion of the transverse sinuses. **(E)** Venogram of right sigmoid sinus, lateral projection after thrombectomy and tPA infusion showing improvement in drainage of the SSS and transverse sinus. Note there is a persistent non-occlusive thrombus of the transverse sinus. **(F)** Axial head CT demonstrating decreased size of the subdural hematoma.

A 69-year-old female presented with COVID-19 pneumonia, altered mental status, atrial fibrillation with rapid ventricular response and severe diarrhea. A head computed tomography (CT) was obtained which showed the initial mixed density acute subdural hematoma (Figure 1A). An additional axial head CT demonstrated multifocal hyperdensities throughout the dural sinuses, highly concerning for multifocal acute dural sinus thrombosis (Figure 1B). She was admitted to the medical intensive care unit (MICU) COVID unit, resuscitated and started on a heparin infusion. Her neurologic course was also complicated by status epilepticus which required intubation, burst suppression and multiple anti-epileptics agents. A CT venogram (CTV) was obtained which confirmed the diagnosis of multi-focal sinus thrombosis. Occlusions were identified in the superior sagittal sinus (SSS), torcula, bilateral transverse sinuses/sigmoid to the jugular bulb and straight sinus along with generalized venous congestion (Figure 1C). While in burst suppression a surveillance head CT was obtained which demonstrated interval development of bilateral hemispheric and posterior fossa mixed density subdural hematomas and generalized sulcus effacement. An optic nerve sheath diameter was obtained, which measured 6 mm, suggestive of intracranial hypertension. The heparin infusion was paused briefly until a definitive treatment plan could be developed.

A multi-disciplinary team discussed multiple treatment options, and ultimately decided with family to resume therapeutic anticoagulation and proceed with endovascular mechanical thrombectomy of the venous sinuses. She was taken for mechanical thrombectomy of SSS and transverse sinuses thrombus with thrombo-aspiration and stent-retriever techniques. Three thrombectomy passes were made using the Solumbra technique with a Trevo 4 × 40 mm stent retriever device, polyvinyl alcohol particles 150–250 microns, and an aspiration catheter. After the third pass, she developed acute desaturations which were concerning for clot mobilization and a pulmonary embolism. The microcatheter was left in the SSS and recombinant tissue plasminogen activator (tPA) was infused for 10 h. The patient's respiratory status quickly improved but several hours after the procedure she developed anisocoria. A head CT was obtained which demonstrated interval expansion of the hemispheric mixed density SDH, global effacement of the sulci and 4th ventricle. The tPA infusion was discontinued. A decision was made to return to the endovascular suite for further attempts at thrombectomy. A total of five thrombectomy passes were completed with removal of large thrombus fragments after each pass.

The final angiogram showed significant improvement in antegrade venous drainage in the SSS, bilateral transverse and sigmoid sinuses with residual multifocal non-occlusive thrombi (Figures 1D,E). After a prolonged hospital course she was discharged to a long term acute care facility. On clinic follow up 10 months later, she had returned living at home independently with a modified ranking score of 2. A head CT obtained during the clinic follow up showed near complete resolution of the SDH (Figure 1F).

### Case 2

**Figure 2:**
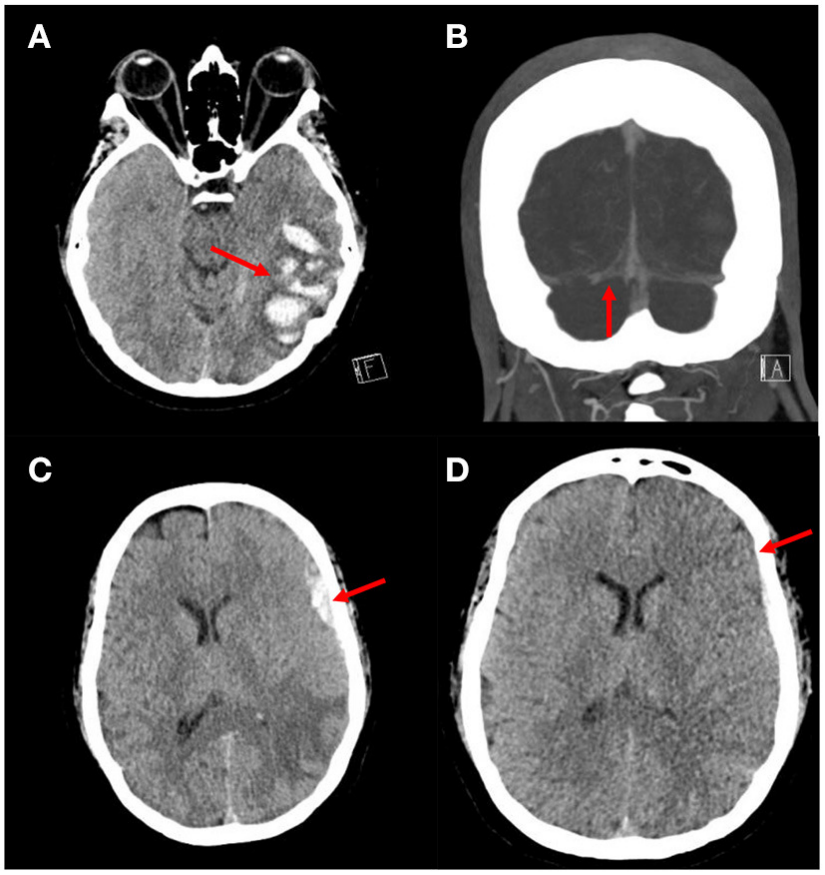
Images from patient who underwent MMA embolization followed by anticoagulation for management of aSDH with CVST. **(A)** Axial head CT demonstrating an ill-defined intraparenchymal hemorrhage in the left temporal lobe concerning for a venous infarct. **(B)** Coronal CTV showing occlusion of the right transverse sinus. **(C)** Axial head CT demonstrating an acute left frontal subdural hematoma. **(D)** Axial head CT demonstrating decreased size of the left frontal subdural hematoma.

A 41-year-old female with history of polysubstance abuse and factor V Leiden mutation who presented with several days of worsening headaches. A head CT was obtained which demonstrated a left temporal intraparenchymal hemorrhage (Figure 2A). She was admitted to the Neuroscience Intensive Care Unit (NSICU) and started on a heparin infusion and then transitioned to dabigatran. A CTV was obtained which demonstrated a thrombosis extending from the straight sinus through the right transverse sinus to the jugular vein (Figure 2B). She was discharged home 7 days after presentation at her neurological baseline. Three days after discharge she presented to the Emergency Department with an acute severe headache and new onset seizures. A head CT demonstrated a new left frontal acute subdural hematoma. The dabigatran was reversed with idarucizumab and she was readmitted to the NSICU. Her neurologic exam remained stable and she was restarted on a heparin infusion.

A surveillance head CT was obtained, which demonstrated a slight interval enlargement of the subdural hematoma (Figure 2C). After discussing the treatment options and need for continued anticoagulation, a decision was reached to proceed with particle embolization of the left middle meningeal artery (MMA). A 5fr Envoy guide catheter, Excelsior 1018 microcatheter and Asahi 14 microwire were used to embolize the left MMA using polyvinyl alcohol 150–250 μm particles. The embolization was uneventful and she tolerated the procedure well. A surveillance head CT obtained 2 days after the embolization demonstrated stable size of the SDH (Figure 2D). She was transitioned from a heparin infusion to dabigatran. She was discharged home 11 days after presentation with no new neurological deficits. At her last clinic follow up 6 months after admission she had returned to living completely independent with a modified ranking score of 0. She is being followed by the hematology department for management of long term anticoagulation.

## Discussion

Cerebral Venous Sinus Thrombosis is a rare cause of stroke accounting for roughly 0.5% of all strokes. CVST frequently affects young adults and pre-menopausal females three times more than males and occurs more often in prothrombotic states such as Factor V Leiden mutation, antithrombin III deficiency, oral contraceptive, trauma, malignancy, and manipulation in operative procedures (1, 2). CVST results in the formation of a thrombus in the cerebral veins, as the thrombus extends, it can occlude draining venous sinuses resulting in a global increased pressure of the venous system. This increased venous pressure can lead to cerebral edema, hemorrhage due to rupture of capillary beds, and a venous infarct (1–3). As a result, the clinical presentation of CVST can be thought of as three separate categories including symptoms of elevated ICP, a focal deficit or a combination of both (1). Generally, headache is the most common presenting symptom and can range from mild to severe. Papilledema can be observed and serves as a surrogate marker for intracranial hypertension. More severe symptoms include altered consciousness, seizures, and focal neurological deficits. Currently, the mainstay treatment for symptomatic CVST is anticoagulation to prevent thrombus extension even in the setting of intraparenchymal hemorrhage (4). However, in severe cases of CVST unresponsive to anticoagulation or in instances where anticoagulation is contraindicated, endovascular thrombolysis or mechanical thrombectomy has been proposed as a possible alternative treatment (1).

Rarely, patients with CVST can develop an aSDH, however, the mechanism of hemorrhage is unclear. Takahashi et al., proposed that a sudden rise in ICP in addition to hemodynamic stress of a CVST can result in the tearing of bridging veins and subsequent formation of aSDH (5). In addition, therapeutic anti-coagulation in CVST, venous hypertension, and the development of spontaneous intracranial hypotension have also been proposed as possible mechanisms for developing an aSDH secondary to CVST (6). Few cases have been reported in the literature that have addressed management approaches to treating aSDH secondary to CVST. Notably, Bansal et al., presented a case of a 40-year-old female on oral contraceptive with an aSDH and subarachnoid hemorrhage secondary to CVST who required a decompressive hemicraniectomy with evacuation of the aSDH. However, as anticoagulation was contraindicated, the patient continued to deteriorate and perished on post-op day one (6). Akins et al. presented a case series of three patients with CVST who developed an aSDH. One patient, a 38-year-old female on oral contraceptive was treated with an intravenous heparin infusion and transitioned to warfarin upon discharge. The second patient was a 68-year-old woman with polycythemia vera who was treated conservatively with hydroxyurea, hydration, and aspirin. The third patient was a 60-year-old male who underwent thrombectomy for CVST that developed 7 days after undergoing burr hole evacuation of an aSDH (2).

Given the complexity of starting or resuming anticoagulation in patients who develop aSDH secondary to CVST, management decisions are difficult. Though it is standard practice to initiate anticoagulation for hemorrhagic venous strokes, this is not well-studied or reported in the literature. Sahoo et al. reported a patient with CVST and aSDH that was successfully managed conservatively with warfarin (7). Furthering management complexity, the pathophysiology of concurrent aSDH and CVST is not well-elucidated and could also be due to different unrelated mechanisms, such as concurrent trauma. In this case report, we present two contrasting cases of management strategies of CVST complicated by development of an aSDH. One patient underwent a mechanical thrombectomy to reduce the overall thrombosis burden and was then managed with anticoagulation and close monitoring. We propose that the thrombectomy was effective in preventing subdural expansion by reducing the global venous pressure and back pressure on the small and fragile bridging veins. In contrast, the second patient was treated with middle meningeal artery (MMA) embolization to prevent subdural hematoma expansion and resumed long-term anticoagulation. Since neither patient had progression or recurrence of the aSDH we propose that MMA embolization or thrombectomy can be considered as a treatment option for CVST complicated by aSDH in the appropriate situation. Further research is necessary to identify appropriate patients and the most efficacious endovascular intervention.

In recent years, embolization of the middle meningeal artery (MMA) has been recognized as an alternative or adjuvant treatment to surgical evacuation for recurrent and primary chronic subdural hematoma (cSDH) (8–12). Notably, peripheral branches of the MMA directly provide the blood supply for fragile sinusoidal neovessels that compose the outer neomembrane of cSDH (9, 10). Embolization of the MMA prevents the recurrent rupture of this neovasculature, which averts hematoma recurrence. This was demonstrated by Onyinzo et al. who analyzed 132 patients who were diagnosed with cSDH and underwent MMA embolization and/or surgical treatment. Patients who were treated with MMA embolization alone received fewer repeat surgeries when compared to those who underwent surgical evacuation. In addition, patients who were treated with MMA embolization were found to have complete hematoma resolution (11). Details regarding the technique of MMA embolization are limited; however, injection of polyvinyl alcohol particles was utilized in our technique and reported by other studies as well (8, 10, 12). Notably, Aronov et al. employed the non-adhesive SQUID-18 embolic agent as a treatment for recurrent cSDH warranting further research to determine the most effective agent (13).

While MMA embolization continues to be a safe and efficacious minimally invasive treatment for cSDH, less is known regarding its efficacy in treating patients who develop aSDH or those who develop aSDH secondary to CVST. Ding et al. successfully performed MMA embolization on a 56-year-old woman with a right hemispheric aSDH 2 days after undergoing a craniotomy for evacuation. The patient did not develop recurrent hemorrhage upon follow-up (12). This was the only study to our knowledge to have explored MMA embolization as a treatment for aSDH in our literature search.

In conclusion, MMA embolization may be a novel approach for management of a patient with aSDH secondary to CVST when anticoagulation is contraindicated. Many unknowns regarding the natural history and pathophysiology of CVST complicated by aSDH are still prevalent. Thus, further research is needed to better establish and understand these mechanisms and to determine whether more aggressive interventional therapies may be beneficial compared to anticoagulation and monitoring alone.

## Data availability statement

The original contributions presented in the study are included in the article/supplementary material, further inquiries can be directed to the corresponding author.

## Ethics statement

Ethical review and approval was not required for the study on human participants in accordance with the local legislation and institutional requirements. Written informed consent for participation was not required for this study in accordance with the national legislation and the institutional requirements. Written informed consent was not obtained from the individual(s) for the publication of any potentially identifiable images or data included in this article.

## Author contributions

NQ, EY, and WL provided direct patient care while the patients were admitted and contributed to the conception and design of the study and wrote sections of the manuscript. MC and NQ reviewed the patient charts and collected the data. MC wrote the first draft of the manuscript. All authors contributed to the manuscript revision, read, and approved the submitted version.

## Conflict of interest

The authors declare that the research was conducted in the absence of any commercial or financial relationships that could be construed as a potential conflict of interest.

## Publisher's note

All claims expressed in this article are solely those of the authors and do not necessarily represent those of their affiliated organizations, or those of the publisher, the editors and the reviewers. Any product that may be evaluated in this article, or claim that may be made by its manufacturer, is not guaranteed or endorsed by the publisher.
